# Retraction Note: Tools for measuring individual self-care capability: a scoping review

**DOI:** 10.1186/s12889-026-28854-4

**Published:** 2026-08-08

**Authors:** Austen El-Osta, Eva Riboli Sasco, Evelina Barbanti, Iman Webber, Aos Alaa, Manisha Karki, Marie line El Asmar, Haitham Idriss, Mashael Almadi, Farah Massoud, Ahmed Alboksmaty, Azeem Majeed

**Affiliations:** Self-Care Academic Research Unit (SCARU), Department of Primary Care & Public Health, School of Public Health, Imperial College London, Charing Cross Hospital, 323 Reynolds BuildingSt Dunstan’s Road, London, W6 8RP UK

**Retraction Note: BMC Public Health 23, 1312** **(2023)**

10.1186/s12889-023-16194-6

The Editors have retracted this article.

Following publication, concerns were raised about the accuracy of the classification, description, and comparison of the self-care measurement tools included in the article. The authors were contacted and acknowledged that some of the concerns are valid. Post-publication peer review identified errors in the definition of key self-care constructs, inaccuracies in the description of instruments such as Self-Care of Chronic Illness Inventory (SCCII), the Self-Care Inventory (SCI) and Self-Care Self-Efficacy Scale (SCSES). These issues affect the reliability of the presented results and undermine the conclusions of this article. The authors were contacted again but were unable to provide a satisfactory response to the concerns raised. The Editors have therefore lost confidence in the data and conclusions presented in this article.

The authors Azeem Majeed and Ahmed Alboksmaty agree with this retraction. The Author Austen El‑Osta disagrees with this retraction. The authors Eva Riboli Sasco, Evelina Barbanti, Iman Webber, Aos Alaa, Manisha Karki, Marie line El Asmar, Haitham Idriss, Mashael Almadi, and Farah Massoud have not responded to correspondence regarding this retraction.

